# Violence, neurocognitive function and clinical correlates in patients with schizophrenia

**DOI:** 10.3389/fpsyt.2022.1087372

**Published:** 2023-01-19

**Authors:** Yun Yi, Yuanyuan Huang, Qiang Chen, Hanlun Yang, Hehua Li, Yangdong Feng, Shixuan Feng, Sumiao Zhou, Zezhi Li, Fengchun Wu

**Affiliations:** ^1^Department of Psychiatry, The Affiliated Brain Hospital of Guangzhou Medical University, Guangzhou, China; ^2^Department of Psychiatry, The Brain Hospital of Guangxi Zhuang Autonomous Region, Guangxi, China; ^3^School of Pharmaceutical Sciences (Shenzhen), Sun Yat-sen University, Shenzhen, China; ^4^Guangdong Engineering Technology Research Center for Translational Medicine of Mental Disorders, Guangzhou, China; ^5^Department of Biomedical Engineering, Guangzhou Medical University, Guangzhou, China

**Keywords:** violence, neurocognitive function, schizophrenia, RBANS, PANSS

## Abstract

**Background:**

Schizophrenia patients with violent behavior are a severe public health concern, but the correlates of this violent behavior are unknown. Additionally, the relationship between neurocognitive function and violent behavior in Chinese patients with schizophrenia has not yet been investigated.

**Methods:**

A total of 337 schizophrenia inpatients were recruited. The Positive and Negative Syndrome Scale (PANSS) was used to assess psychopathological symptoms. Neurocognitive functioning was evaluated using the Repeatable Battery for the Assessment of Neuropsychological Status (RBANS).

**Results:**

The percentage of violent behavior was 10.4% in patients with schizophrenia. Patients with violent behavior had higher PANSS-positive, excited, and total subscale scores than patients who did not show violent behavior. Patients with violent behavior also had lower RBANS language, semantic fluency, and total subscale scores. Gender (OR = 0.066∼0.819, *p* = 0.023), illness duration (OR = 0.876∼0.971, *p* = 0.002), smoking (OR = 1.127∼2.950, *p* = 0.014), the PANSS positive subscale (OR = 1.050∼1.197, *p* = 0.001), and the RBANS language subscale (OR = 0.927∼0.987, *p* = 0.005) significantly contributed to the development of violent behavior in schizophrenia patients.

**Conclusion:**

Our findings revealed that cognitive and clinical assessments should be considered in comprehensive assessments of future risks of violence in schizophrenia patients.

## Introduction

Numerous studies have found that there exists an association between schizophrenia and the increasing risk of violent behavior (1–3). It is reported that patients with schizophrenia are at a higher risk of violence than the general population (4, 5). Patients with schizophrenia have a higher proportion in murderers, accounting for 5–20% of all homicide offenders (6). Violent behavior can cause more frequent and longer hospitalization for patients, which increases the utilization and expense of medical services (7).

In fact, apart from a small minority of patients with schizophrenia, most of them are not chronically violent (8). Further, patients with schizophrenia are more susceptible to be the victims of violence (9, 10). Unfortunately, the excessive reinforcement of violence by the news media has led to the negative views of the public on such people, which increase the awareness of the risk of mental illness and contributes to stigmatization (11). Some studies focusing on the association may enhance the perception of the public (12). Therefore, it is challengeable to investigate the association between schizophrenia and violence. However, denying the association would fail to meet the clinical needs of psychiatric services (12). Therefore, it needs a balanced study of violent behaviors in patients with schizophrenia to develop treatment and prevention programs targeted at reducing such behaviors.

Previous studies have shown that various demographic characteristics are associated with violent behavior in schizophrenia, including: male sex (13, 14), non-adherence to treatment (15), substance abuse (1, 2, 16–18), younger age (13), history of violence (2, 15, 19), personality disorders (20), and childhood trauma (21–23). Clinical risk factors of violent behavior in patients with schizophrenia are anxiety (19), poor sleep quality (24, 25), hallucination and delusion (26, 27), poor insight (26, 28), and impulsivity (29). However, conclusions are still inconsistent. For example, a study of 7,711 schizophrenia patients revealed that delusions of percussion and auditory hallucination decreased the risk of violence, while destruction of the property, verbal aggression, and insomnia increased the risk of violent behavior (24). It needs further study to investigate the relationship between the demographic factors and violence behavior.

In addition, recent studies have shown that impairments in cognitive functioning can increase the risk of violent behavior (30, 31), and patients with schizophrenia with violent behavior have defects in both general intellectual ability and specific cognitive functions (32–34). Hsu and Ouyang (35) showed that the increased neurocognitive scores were significantly associated with decreased risks of violent behavior. Some studies also showed that a combination of neurocognitive and social-cognitive treatment could reduce violence (35–37). However, the patterns of specific cognitive impairment were inconsistent across these studies. For example, Ahmed et al. (34) found that violent behaviors in schizophrenia are associated with deficits in working memory, verbal learning, and reasoning/problem solving. Serper et al. (38) showed that altered executive functioning in schizophrenia patients may lead to a lack of reasoning and lack of behavioral inhibition, which can eventually evolve into violence. However, other evidence has shown that schizophrenia patients with antisocial or violent behaviors have less executive deficiency than patients without these behaviors (39–41). Furthermore, the relationship between violent behavior and neurocognitive functioning in Chinese patients with schizophrenia has not been investigated.

Therefore, the aim of this study was to investigate: (1) the percentage of violence in Chinese inpatients with schizophrenia; (2) factors correlating with risk of violence in Chinese patients with schizophrenia, if any; (3) whether neurocognitive functioning associated with violent behaviors in Chinese patients with schizophrenia.

## Materials and methods

### Subjects

This study was approved by the Institutional Review Board of the Affiliated Brain Hospital of Guangzhou Medical University, and all the study procedures were in accordance with the Declaration of Helsinki. A total of 337 inpatients (at least 1-month hospitalization) were recruited from this institute between March 2018 and September 2019. Each participant signed informed consent forms. The inclusion criteria were as follows: (1) aged between 18 and 50 years; (2) satisfied the diagnosis of schizophrenia according to the DSM-IV-TR; and (3) member of the Chinese Han population. The exclusion criteria were as follows: (1) diagnosis of other neurological or any severe physical diseases; (2) diagnosis of any other major Axis I disorder; (3) diagnosis of substances abuse or alcohol dependence; (4) receipt of MECT (Modified Electric Convulsive Treatment) treatment in the past 6 months; and (5) pregnancy or lactation.

### Demographic and clinical information

Socio-demographic and clinical information was collected through self-designed questionnaires and medical records. Violence behavior can be described as aggression toward property or physical aggression in our study. Aggression toward property refers to a deliberate behavior of destruction of property. Physical aggression refers to the behavior of causing pain, physical injury, or death to others (42). All participants were asked the following question: “Have you engaged in any of the following violent activities in the hospitalization: aggression toward property or physical aggression toward others?” (Violent behavior was defined as an affirmative answer in at least one of the two situations) (43). We would use secondary sources (e.g., records, staff) serving as collateral databases for each patient’s history of violence relative to the requirements. We have also indicated the subjects’ report of violent behavior corresponded to the medical records account of violence.

### Clinical and cognitive measurements

All participants were screened by two experienced psychiatrists, who confirmed the diagnosis of schizophrenia through the Structured Clinical Interview for DSM-IV (SCID-I/P) (SCID). The Positive and Negative Syndrome Scale (PANSS) was used to assess psychopathological symptoms (44). In this study, we used a five-factor model of PANSS as follows (45): positive subscale (P1, P3, P5, and G9), negative subscale (N1, N2, N3, N4, N6, G7), excitement subscale (P4, P7, G8, G14), depression and anxiety subscale (G2, G3, G6), and cognitive subscale (P2, N5, G11).

A better established of its clinical validity and test–retest reliability, the Chinese version of the Repeatable Battery for the Assessment of Neuropsychological Status (RBANS), was utilized to value the neurocognitive functioning of patients (46). The RBANS contains five dimensions and 12 subtests. The battery test includes the following subtests: immediate memory (list learning and story memory tasks), attention (digit span and coding tasks), language (picture naming and semantic fluency tasks), visuospatial/construction (figure copy and line orientation tasks), and delayed memory (list recall, story recall, figure recall, and list recognition tasks). Generally, it took about 30–60 min to complete the tests.

To maintain the consistency and reliability of rating, all psychiatrists who enrolled in this study simultaneously accepted a training course for standardizing their use in SCID, PANSS, and RBANS assessments ahead of the study. After training, the psychiatrists of the inter-observer correlation coefficient (ICC) were greater than 0.8, higher than the critical point proposed by Shrout and Fleiss (47).

### Statistical analysis

A sample Kolmogorov–Smirnov test was used to determine whether the continuous data were normally distributed. Demographic and clinical variables in both groups were compared using analysis of variance for continuous variables and chi-squared tests for categorical variables. When there were significant differences in the analysis of variance, analysis of covariance (ANCOVA) was performed by controlling for covariates. We also conducted binary logistic regression models (forward: conditional model) to examine factors that could potentially have contributed to violent behavior in patients with schizophrenia. Statistical analysis was conducted using SPSS25.0. The significance level was set to 0.05 (two-tailed).

## Results

### Demographic and clinical characteristics

Demographic and clinical characteristics are shown in Table 1. A total of 337 schizophrenia patients (male = 222, female = 115; mean age = 38.76 ± 7.85) were recruited. Of the 337 schizophrenia patients, 35 (10.4%) had violent behavior. Compared to the non-violence group, the violence group had older age of disease onset (*F* = 6.383, *p* < 0.05) and shorter illness durations (*F* = 9.716, *p* < 0.05). Male patients were more likely to exhibit violent behavior than female patients (χ^2^ = 6.715, *p* = 0.01). The rate of current smoking in the violence group (19/35) was higher than that in the non-violence group (χ^2^ = 12.586, *p* < 0.001).

**TABLE 1:** Demographic characteristics of violent and non-violent patients.

	Non-violence (*n* = 302)	Violence (*n* = 35)	*F*/χ^2^	*p*-value
Age	38.89 ± 7.91	36.97 ± 7.58	1.858	0.174
Years of education	8.95 ± 2.96	9.03 ± 3.02	0.022	0.883
BMI	25.10 ± 4.40	25.79 ± 5.17	0.756	0.385
Onset age	23.16 ± 6.06	25.97 ± 7.68	6.383	0.012*
Illness duration	15.73 ± 8.58	11 ± 7.79	9.716	0.002**
Numbers of hospitalization	6.73 ± 8.27	6.44 ± 8.15	0.037	0.849
Antipsychotic drug dosage	291.64 ± 262.91	220.59 ± 167.29	2.386	0.123
(CPZ equivalent mg), M ± SD				
Female/male	110/194	5/30	6.715	0.01*
**The status of smoking, *n* (%)**				
Non-smoking	218 (75.3)	16 (45.7)	12.586	<0.001**
Now smoking	75 (24.7)	19 (54.3)		
**Marriage status, *n* (%)**				
Unmarried	203 (66.8)	25 (71.4)	2.610	0.456
Married	55 (18.1)	4 (11.4)		
Divorced	43 (14.1)	5 (14.3)		
Widowed	2 (0.7)	1 (2.9)		

BMI, body mass index. *p < 0.05, **p < 0.005.

### Clinical characteristics in violent patients and non-violent patients

As shown in Table 2, the violence group had higher scores on the PANSS positive subscale, the excited subscale, the cognitive subscale, and the total subscale (all *p*’s < 0.05). After Bonferroni corrections, the PANSS positive subscale (*p*’s _*Bonferroni*_ = 0.012), the cognitive subscale (*p*’s _*Bonferroni*_ = 0.03), and the total subscale (*p*’s _*Bonferroni*_ = 0.024) were passed the correction, excepted for excited subscale (*p*’s _*Bonferroni*_ = 0.06). After adjusting for gender, onset age, and smoking status, the violence group still had higher scores on the PANSS positive subscale (*F* = 11.876, *p* = 0.001), PANSS excited subscale (*F* = 6.284, *p* = 0.013), PANSS cognitive subscale (*F* = 10.046, *p* = 0.002), and PANSS total (*F* = 12.756, *p* < 0.01).

**TABLE 2:** Clinical variables and cognitive functions between violent and non-violent patients.

	Non-violence *n* = 302, M ± SD	Violence *n* = 35, M ± SD	*F*	*p*-value
PANSS total score	73.35 ± 16.86	84.14 ± 16.59	8.565	0.004**
Positive subscore	16.02 ± 5.36	19.17 ± 6.72	10.241	0.002**
Negative subscore	20.28 ± 6.6	21.43 ± 7.21	0.933	0.335
Excited subscore	7.74 ± 2.89	9.11 ± 3.47	6.735	0.01*
Cognitive subscore	10.68 ± 2.98	12.20 ± 3.52	7.892	0.005*
Depressive subscore	7.32 ± 2.67	7.89 ± 3.00	1.375	0.242
RBANS total score	66.4 ± 14.2	61.1 ± 11.4	4.559	0.033*
Immediate memory	59.80 ± 32.73	54.14 ± 14.47	1.020	0.313
Visuospatial/ constructional	79.36 ± 18.32	73.57 ± 12.15	3.324	0.069
Language	80.92 ± 14.15	75.11 ± 12.49	5.413	0.021*
Attention	79.37 ± 15.51	76.49 ± 14.96	1.092	0.297
Delayed memory	65.97 ± 19.95	60.34 ± 20.45	2.489	0.116

PANSS, Positive and Negative Syndrome Scale; RBANS, Repeatable Battery for the Assessment of Neuropsychological Status. *p < 0.05, **p < 0.005.

### Cognitive function in violent patients and non-violent patients

As shown in Table 2, there were significant differences in Language and RBANS total score between the two groups (*p* < 0.05). When adjusting for gender, onset age, and years of education, the violence group had lower Language (*F* = 8.536, *p* = 0.004) and RBANS total (*F* = 9.539, *p* = 0.002) scores than the non-violence group.

### Correlation of violent behavior, clinical symptoms, and cognitive performance

Binary logistic regression analysis was used to examine the associated factors that contributed to violent behavior in schizophrenia patients. The following variables were set as independent factors: gender, age of disease onset, duration of illness, smoking status, PANSS positive factor, PANSS excited factor, PANSS cognitive factor, PANSS total factor, RBANS Language subscale, and total score. As shown in Table 3, gender (OR = 0.233, 95% CI: 0.066–0.819), duration of illness (OR = 0.923, 95% CI: 0.876–0.971), current status smoking (OR = 1.824, 95% CI: 1.127–2.950), PANSS-positive subscale (OR = 1.121, 95% CI: 1.050–1.197), and RBANS Language scale (OR = 0.956, 95% CI: 0.927–0.987) were independently associated with violent behavior in patients with schizophrenia.

**TABLE 3:** Factors for violent behavior in schizophrenia.

	B	Odds ratio (OR)	95% CI		*p*-value
			**Lower**	**Upper**	
Illness duration	–0.081	0.923	0.876	0.971	0.002
Gender	–1.459	0.233	0.066	0.819	0.023
Status of smoking	0.601	1.824	1.127	2.950	0.014
PANSS positive	0.114	1.121	1.050	1.197	0.001
Language	–0.045	0.956	0.927	0.987	0.005

Variables in the model: Age of onset, Duration of illness, Gender, the status of smoking, PANSS total score, Positive subscore, Excited subscore, Cognitive subscore, RBANS total score, and Language.

## Discussion

The main results of this study were as follows: (1) the percentage of violence in patients with schizophrenia was 10.4%; (2) patients with schizophrenia with violent behavior performed worse on the RBANS-list language and total scales than those without violent behavior; and (3) male sex, shorter duration of illness, smoking, scores of the positive PANSS, and RBANS-list language scales were associated with violent behavior in patients with schizophrenia.

The percentage of violent behavior amongst our cohort was similar to previous studies (1, 48). However, the percentage of violent behavior among patients with schizophrenia varied widely across previous studies. Zhou et al. (49) conducted a meta-analysis on 3941 patients in China and reported that at least 31.6% of them exhibited violent behavior during the hospitalization. Swanson et al. (50) reported the percentage of any violence in 1410 schizophrenia patients was about 19.1% over 6 months. Knezevic et al. (51) showed that the prevalence of violent behavior in 1,330 Serbian schizophrenia outpatients was 31%. The possible reasons for these discrepancies may be differing criteria for violence, different disease stages (acute, early, active phase, or remission) (51), the type of antipsychotic drugs taken, and different ethnic origins or genetic backgrounds of study participants.

The present study revealed that violent patients had lower RBANS total scores, as well as lower RBANS language than patients exhibiting non-violent behavior. Furthermore, the RBANS-language score was significantly correlated with violent behavior in patients with schizophrenia. An alternative explanation of the findings is that the violent group may be experiencing more positive symptoms that negatively impact their neuro-cognitive performance on the RBANS. However, previous evidence demonstrated that neurocognitive functioning decline was considerate to occur ahead of the onset of psychosis in patients with schizophrenia (52, 53). It was reasonable to point out that neurocognitive functioning had an impact on psychosis symptoms rather than the other way around.

Our results indicated that poor neurocognitive functioning was associated with the increasing risk of violent behavior in patients with schizophrenia, which was consistent with several previous results (30, 34). In the clinic, the neuropsychological assessment tools like verbal fluency tests would be used to value the executive function (EF) (54, 55). Therefore, we would like to show potential underlying mechanisms from the insight of EF to clarify the association between cognitive deficit and violence behavior. First, cognitive reappraisal is an emotional regulation strategy, which can explain situations that may provoke emotions in a way that affects the impact of emotion (56). Reappraisal requires proper executive functioning (57), as well as the expression of positive emotion. However, deficits in cognitive function result in failures of emotional reappraisal, which prevent emotion from being properly processed and expressed, increasing the likelihood of violence. Second, cognitive decline may contribute to the development of ineffective coping strategies and the loss of automatic motor response inhibition, which can reduce the ability to self-regulate anger (37). Third, patients with executive dysfunction may not be able to inhibit their behavior properly (58). Patients with schizophrenia need to cope with their symptoms and other stressors related to acute psychosis and hospitalization, which may increase violent behavior. Moreover, EF impairment can cause hostile attribution bias through the misunderstanding of real clues and failure to update and modify cognitive processes accordingly (59). Additionally, Sematic fluency test is also utilized to value the ability to organize thought (60). Therefore, the lower of semantic fluency means the poor ability of thought organization, which may be correlated with the violence behavior. However, there have been some inconsistent conclusions in the literature. Kashiwagi et al. (39) recruited 54 patients and showed that those exhibiting violence had significantly better EF than those who did not exhibit violence. Bulgari et al. (41) compared patients with schizophrenia in Residential Facilities with histories of violent behavior (*n* = 50) to schizophrenia patients without histories of violence (*n* = 37) and showed that the group exhibiting violence performed better on executive and motor tasks. The differing conclusions may also be explained by inconsistent definitions of violence and divergent relative sample sizes.

Our study showed that male patients with schizophrenia were more likely to display violent behavior, consistent with previous studies (13, 14). This may be due to physiological differences (e.g., Greater testosterone levels). Men have been shown to be 12.8 times more like to commit violent behavior than women (61). Our study discovered a substantial negative association between illness duration and violent behavior. The underlying reason may be that patients with longer illness durations exhibited differing fundamental characteristics and negative symptoms, including emotional bluntness, decreased activity, social isolation, and reduced communication capacities, all of which are correlated with lower risks of violence (41).

Our results demonstrated that PANSS positive subscale scores were associated with violent behavior in schizophrenia patients, which mirrors previous results (62, 63). This finding could potentially be explained by the notion that the occurrence and frequency of hallucination and delusion may be associated with deficits of intention control in invasive cognitive function (64) because hallucinations elicited unpleasant feelings (anger, grief) that the patients were unable to cope with. Interestingly, we also found that smoking contributed to the increase in violence, which was also in line with previous studies (61, 65, 66). One study showed that nicotine replacement therapy could reduce agitation and aggression in patients with schizophrenia who smoke (67). Nicotine deprivation and withdrawal due to long hospital stays can also increase the risk for impulsivity (68). Patients expressed dissatisfaction that they were not permitted to smoke or obtain nicotine. This study shows that we should not overlook violent behaviors that result from substance abuse in our clinical work.

There are several limitations to our study that should be considered. First, because of the cross-sectional nature of our study, we were only able to demonstrate a link between clinical characteristics, neurocognitive factors, and violence. Future prospective studies should be conducted to clarify how these variables contribute to the development of violent issues in schizophrenia patients. Second, other dynamic factors may affect violent behaviors. These could be neurocognitive factors, such as social support or emotional levels expressed by caregivers. Third, some factors that may affect the results were not considerate as covariates, including medication history, childhood trauma, the state of sleeping, and any other factors associated with violence. Forth, since we did not recruit healthy controls for comparison, the impacts of result implications should be interpreted carefully. Fifth, in our study, we didn’t identify questions as indices of violence. We would design a short-form measure to identify the indices of violence in the further study.

## Conclusion

In conclusion, cognitive and clinical assessments should be incorporated into comprehensive assessments related to future risks of violence in patients with schizophrenia. This could help identify patients who are at risk of becoming violent and inform the creation of personalized interventions.

## Data availability statement

The original contributions presented in this study are included in this article/supplementary material, further inquiries can be directed to the corresponding authors.

## Ethics statement

The studies involving human participants were reviewed and approved by the Affiliated Brain Hospital of Guangzhou Medical University. The patients/participants provided their written informed consent to participate in this study.

## Author contributions

FW and ZL designed the study. HL, YF, SF, and SZ screened the patients and collected the data. QC and HY collected the literature and cleaned the data. YY and YH performed the statistical analysis. YY, YH, ZL, and FW wrote the manuscript. All authors discussed the results and reviewed the manuscript.

## References

[1] Fazel S Gulati G Linsell L Geddes JR Grann M. Schizophrenia and violence: systematic review and meta-analysis. *PLoS Med.* (2009) 6:e1000120. 10.1371/journal.pmed.1000120 19668362 PMC2718581

[2] Fazel S Wolf A Palm C Lichtenstein P. Violent crime, suicide, and premature mortality in patients with schizophrenia and related disorders: a 38-year total population study in Sweden. *Lancet Psychiatry.* (2014) 1:44–54. 10.1016/s2215-0366(14)70223-8 25110636 PMC4124855

[3] Fleischman A Werbeloff N Yoffe R Davidson M Weiser M. Schizophrenia and violent crime: a population-based study. *Psychol Med.* (2014) 44:3051–7. 10.1017/s0033291714000695 25065575

[4] Fazel S Långström N Hjern A Grann M Lichtenstein P. Schizophrenia, substance abuse, and violent crime. *JAMA.* (2009) 301:2016–23. 10.1001/jama.2009.675 19454640 PMC4905518

[5] Nolan KA D’Angelo D Hoptman MJ. Self-report and laboratory measures of impulsivity in patients with schizophrenia or schizoaffective disorder and healthy controls. *Psychiatry Res.* (2011) 187:301–3. 21106252 10.1016/j.psychres.2010.10.032 PMC3075418

[6] Nielssen O Large M. Rates of homicide during the first episode of psychosis and after treatment: a systematic review and meta-analysis. *Schizophr Bull.* (2010) 36:702–12. 10.1093/schbul/sbn144 18990713 PMC2894594

[7] Zhang J Harvey C Andrew C. Factors associated with length of stay and the risk of readmission in an acute psychiatric inpatient facility: a retrospective study. *Aust N Z J Psychiatry.* (2011) 45:578–85. 10.3109/00048674.2011.585452 21718126 PMC3190839

[8] Strassnig MT Nascimento V Deckler E Harvey PD. Pharmacological treatment of violence in schizophrenia. *CNS Spectrums.* (2020) 25:207–15. 10.1017/s1092852919001226 31342892

[9] Maniglio R. Severe mental illness and criminal victimization: a systematic review. *Acta Psychiatr Scand.* (2009) 119:180–91. 10.1111/j.1600-0447.2008.01300.x 19016668

[10] El Missiry A Meguid MAE Abourayah A Missiry ME Hossam M Elkholy H Rates and profile of victimization in a sample of Egyptian patients with major mental illness. *Int J Soc Psychiatry.* (2019) 65:183–93. 10.1177/0020764019831315 30848686

[11] Lok P Dijk S. Violence and mental illness: five minutes with seena Fazel. *BMJ.* (2019) 366:l5199. 10.1136/bmj.l5199 31420349

[12] Whiting D Gulati G Geddes JR Fazel S. Association of schizophrenia spectrum disorders and violence perpetration in adults and adolescents from 15 countries: a systematic review and meta-analysis. *JAMA Psychiatry.* (2022) 79:120–32. 10.1001/jamapsychiatry.2021.3721 34935869 PMC8696689

[13] Dack C Ross J Papadopoulos C Stewart D Bowers LA. review and meta-analysis of the patient factors associated with psychiatric in-patient aggression. *Acta Psychiatr Scand.* (2013) 127:255–68. 10.1111/acps.12053 23289890

[14] Hachtel H Harries C Luebbers S Ogloff JR. Violent offending in schizophrenia spectrum disorders preceding and following diagnosis. *Aust N Z J Psychiatry.* (2018) 52:782–92. 10.1177/0004867418763103 29543067

[15] Buchanan A Sint K Swanson J Rosenheck R. Correlates of future violence in people being treated for schizophrenia. *Am J Psychiatry.* (2019) 176:694–701. 10.1176/appi.ajp.2019.18080909 31014102

[16] Lamsma J Cahn W Fazel S. Use of illicit substances and violent behaviour in psychotic disorders: two nationwide case-control studies and meta-analyses. *Psychol Med.* (2020) 50:2028–33. 10.1017/s0033291719002125 31462346 PMC7525769

[17] Kalk NJ Robins JE Ross KR Pritchard M Lynskey MT Curtis VA Substance use in psychiatric crisis: relationship to violence. *Psychol Med.* (2020) 52:1691–7. 10.1017/s0033291720003451 33148358

[18] Beaudoin M Potvin S Giguère CE Discepola SL Dumais A. Persistent cannabis use as an independent risk factor for violent behaviors in patients with schizophrenia. *NPJ Schizophrenia.* (2020) 6:14. 10.1038/s41537-020-0104-x 32393793 PMC7214412

[19] Wang QW Hou CL Wang SB Huang ZH Huang YH Zhang JJ Frequency and correlates of violence against patients with schizophrenia living in rural China. *BMC Psychiatry.* (2020) 20:286. 10.1186/s12888-020-02696-9 32505208 PMC7275550

[20] Candini V Ghisi M Bottesi G Ferrari C Bulgari V Iozzino L Personality, schizophrenia, and violence: a longitudinal study. *J Personal Disord.* (2018) 32:465–81. 10.1521/pedi_2017_31_304 28758886

[21] Faay MDM van Os J. Aggressive behavior, hostility, and associated care needs in patients with psychotic disorders: a 6-year follow-up study. *Front Psychiatry.* (2019) 10:934. 10.3389/fpsyt.2019.00934 31998154 PMC6961536

[22] Fosse R Eidhammer G Selmer LE Knutzen M Bjørkly S. Strong associations between childhood victimization and community violence in male forensic mental health patients. *Front Psychiatry.* (2020) 11:628734. 10.3389/fpsyt.2020.628734 33633598 PMC7901946

[23] Oakley C Harris S Fahy T Murphy D Picchioni M. Childhood adversity and conduct disorder: a developmental pathway to violence in schizophrenia. *Schizophr Res.* (2016) 172:54–9. 10.1016/j.schres.2016.01.047 26879586

[24] Sun L Han X Wang K Xu C Song Z Zhang J Candidate symptomatic markers for predicting violence in schizophrenia: a cross-sectional study of 7711 patients in a Chinese population. *Asian J. Psychiatr.* (2021) 59:102645. 10.1016/j.ajp.2021.102645 33845298

[25] Chen ZT Wang HT Chueh KH Liu IC Yang CM. An exploration of the sleep quality and potential violence among patients with schizophrenia in community. *Perspect Psychiatr Care.* (2021) 57:648–54. 10.1111/ppc.12589 32730660

[26] Rund BR. A review of factors associated with severe violence in schizophrenia. *Nordic J Psychiatry.* (2018) 72:561–71. 10.1080/08039488.2018.1497199 30099913

[27] Amr M Elsayed H Ibrahim IM. Impulsive behavior and its correlates among patients with schizophrenia in a tertiary care psychiatry setting in Mansoura. *Asian J Psychiatr.* (2016) 22:111–5. 10.1016/j.ajp.2016.06.009 27520910

[28] Witt K van Dorn R Fazel S. Risk factors for violence in psychosis: systematic review and meta-regression analysis of 110 studies. *PLoS One.* (2013) 8:e55942. 10.1371/journal.pone.0055942 23418482 PMC3572179

[29] Moulin V Golay P Palix J Baumann PS Gholamrezaee MM Azzola A Impulsivity in early psychosis: a complex link with violent behaviour and a target for intervention. *Eur Psychiatry.* (2018) 49:30–6. 10.1016/j.eurpsy.2017.12.003 29353178

[30] Reinharth J Reynolds G Dill C Serper M. Cognitive predictors of violence in schizophrenia: a meta-analytic review. *Schizophr Res Cogn.* (2014) 1:101–11. 10.1016/j.scog.2014.06.001

[31] O’Reilly K Donohoe G Coyle C O’Sullivan D Rowe A Losty M Prospective cohort study of the relationship between neuro-cognition, social cognition and violence in forensic patients with schizophrenia and schizoaffective disorder. *BMC Psychiatry.* (2015) 15:155. 10.1186/s12888-015-0548-0 26159728 PMC4496853

[32] Reilly JL Sweeney JA. Generalized and specific neurocognitive deficits in psychotic disorders: utility for evaluating pharmacological treatment effects and as intermediate phenotypes for gene discovery. *Schizophr Bull.* (2014) 40:516–22. 10.1093/schbul/sbu013 24574307 PMC3984526

[33] Stratton J Cobia DJ Reilly J Brook M Hanlon RE. Differences in neuropsychological functioning between homicidal and nonviolent schizophrenia samples. *J Forensic Sci.* (2018) 63:1435–43. 10.1111/1556-4029.13750 29411382

[34] Ahmed AO Richardson J Buckner A Romanoff S Feder M Oragunye N Do cognitive deficits predict negative emotionality and aggression in schizophrenia? *Psychiatry Res.* (2018) 259:350–7. 10.1016/j.psychres.2017.11.003 29120842

[35] Hsu MC Ouyang WC. Effects of integrated violence intervention on alexithymia, cognitive, and neurocognitive features of violence in schizophrenia: a randomized controlled trial. *Brain Sci.* (2021) 11:837. 10.3390/brainsci11070837 34202608 PMC8301770

[36] Jones MT Harvey PD. Neurocognition and social cognition training as treatments for violence and aggression in people with severe mental illness. *CNS Spectrums.* (2020) 25:145–53. 10.1017/s1092852919001214 31248468

[37] Darmedru C Demily C Franck N. Cognitive remediation and social cognitive training for violence in schizophrenia: a systematic review. *Psychiatry Res.* (2017) 251:266–74. 10.1016/j.psychres.2016.12.062 28219026

[38] Serper M Beech DR Harvey PD Dill C. Neuropsychological and symptom predictors of aggression on the psychiatric inpatient service. *J Clin Exp Neuropsychol.* (2008) 30:700–9. 10.1080/13803390701684554 18608673

[39] Kashiwagi H Kuroki N Ikezawa S Matsushita M Ishikawa M Nakagome K Neurocognitive features in male patients with schizophrenia exhibiting serious violence: a case control study. *Ann General Psychiatry.* (2015) 14:46. 10.1186/s12991-015-0086-7 26697100 PMC4687370

[40] Naudts K Hodgins S. Neurobiological correlates of violent behavior among persons with schizophrenia. *Schizophr Bull.* (2006) 32:562–72. 10.1093/schbul/sbj036 16384876 PMC2632244

[41] Bulgari V Iozzino L Ferrari C Picchioni M Candini V De Francesco A Clinical and neuropsychological features of violence in schizophrenia: a prospective cohort study. *Schizophr Res.* (2017) 181:124–30. 27765521 10.1016/j.schres.2016.10.016

[42] Allen DE Mistler LA Ray R Batscha C Delaney K Loucks J Call to action from the APNA council for safe environments: defining violence and aggression for research and practice improvement purposes. *J Am Psychiatr Nurses Assoc.* (2019) 25:7–10. 10.1177/1078390318809159 30394822

[43] Krug EG Mercy JA Dahlberg LL Zwi AB. The world report on violence and health. *Lancet.* (2002) 360:1083–8. 10.1016/s0140-6736(02)11133-0 12384003

[44] Kay SR Fiszbein A Opler LA. The positive and negative syndrome scale (PANSS) for schizophrenia. *Schizophr Bull.* (1987) 13:261–76. 10.1093/schbul/13.2.261 3616518

[45] Wallwork RS Fortgang R Hashimoto R Weinberger DR Dickinson D. Searching for a consensus five-factor model of the positive and negative syndrome scale for schizophrenia. *Schizophr Res.* (2012) 137:246–50. 10.1016/j.schres.2012.01.031 22356801 PMC3351536

[46] Zhang BH Tan YL Zhang WF Wang ZR Zhou DF. Repeatable battery for the assessment of neuropsychological status (RBANS) as a screening test in Chinese: reliability and validity. *J Chinese Mental Health.* (2008) 22:865–9.

[47] Shrout PE Fleiss JL. Intraclass correlations: uses in assessing rater reliability. *Psychol Bull.* (1979) 86:420–8. 10.1037//0033-2909.86.2.420 18839484

[48] Randolph C Tierney MC Mohr E Chase TN. The repeatable battery for the assessment of neuropsychological status (RBANS): preliminary clinical validity. *J Clin Exp Neuropsychol.* (1998) 20:310–9. 10.1076/jcen.20.3.310.823 9845158

[49] Zhou JS Zhong BL Xiang YT Chen Q Cao XL Correll CU Prevalence of aggression in hospitalized patients with schizophrenia in China: a meta-analysis. *Asia-Pacific Psychiatry.* (2016) 8:60–9. 10.1111/appy.12209 26346165

[50] Swanson JW Swartz MS Van Dorn RA Elbogen EB Wagner HR Rosenheck RA national study of violent behavior in persons with schizophrenia. *Arch Gen Psychiatry.* (2006) 63:490–9. 10.1001/archpsyc.63.5.490 16651506

[51] Knezevic V Mitrovic D Drezgic-Vukic S Knezevic J Ivezic A Siladji-Mladenovic D Prevalence and correlates of aggression and hostility in hospitalized schizophrenic patients. *J Interpersonal Viol.* (2017) 32:151–63. 10.1177/0886260515585537 26037811

[52] Kahn RS Keefe RS. Schizophrenia is a cognitive illness: time for a change in focus. *JAMA Psychiatry.* (2013) 70:1107–12. 10.1001/jamapsychiatry.2013.155 23925787

[53] Soyka M. Neurobiology of aggression and violence in schizophrenia. *Schizophr Bull.* (2011) 37:913–20. 10.1093/schbul/sbr103 21860037 PMC3160223

[54] Chan RC Shum D Toulopoulou T Chen EY. Assessment of executive functions: review of instruments and identification of critical issues. *Arch Clin Neuropsychol.* (2008) 23:201–16. 10.1016/j.acn.2007.08.010 18096360

[55] Faria CA Alves HVD Charchat-Fichman H. The most frequently used tests for assessing executive functions in aging. *Dement Neuropsychol.* (2015) 9:149–55. 10.1590/1980-57642015dn92000009 29213956 PMC5619353

[56] Gross JJ John OP. Individual differences in two emotion regulation processes: implications for affect, relationships, and well-being. *J Pers Soc Psychol.* (2003) 85:348–62. 10.1037/0022-3514.85.2.348 12916575

[57] Lantrip C Isquith PK Koven NS Welsh K Roth RM. Executive function and emotion regulation strategy use in adolescents. *Appl Neuropsychol. Child.* (2016) 5:50–5. 10.1080/21622965.2014.960567 25650638

[58] Weiss EM. Neuroimaging and neurocognitive correlates of aggression and violence in schizophrenia. *Scientifica.* (2012) 2012:158646. 10.6064/2012/158646 24278673 PMC3820648

[59] Krakowski MI Czobor P. Executive function predicts response to antiaggression treatment in schizophrenia: a randomized controlled trial. *J Clin Psychiatry.* (2012) 73:74–80. 10.4088/JCP.11m07238 22152404

[60] Strauss E Sherman EMS Spreen O. *A Compendium of Neuropsychological Tests: Administration, Norms, and comMentary.* 3rd ed. New York, NY: Oxford University Press (2006).

[61] Lejoyeux M Nivoli F Basquin A Petit A Chalvin F Embouazza H. An investigation of factors increasing the risk of aggressive behavior among schizophrenic inpatients. *Front Psychiatry.* (2013) 4:97. 10.3389/fpsyt.2013.00097 24027539 PMC3759799

[62] Coid JW Ullrich S Kallis C Keers R Barker D Cowden F The relationship between delusions and violence: findings from the East London first episode psychosis study. *JAMA Psychiatry.* (2013) 70:465–71. 10.1001/jamapsychiatry.2013.12 23467760

[63] Keers R Ullrich S Destavola BL Coid JW. Association of violence with emergence of persecutory delusions in untreated schizophrenia. *Am J Psychiatry.* (2014) 171:332–9. 10.1176/appi.ajp.2013.13010134 24220644

[64] Li P Fan TT Zhao RJ Han Y Shi L Sun HQ Altered brain network connectivity as a potential endophenotype of schizophrenia. *Sci Rep.* (2017) 7:5483. 10.1038/s41598-017-05774-3 PMC5511161 28710394

[65] Parrott DJ Zeichner A. Effects of nicotine deprivation and irritability on physical aggression in male smokers. *Psychol Addict Behav.* (2001) 15:133–9. 11419229

[66] Kao YC Liu YP Cheng TH Chou MK. Cigarette smoking in outpatients with chronic schizophrenia in Taiwan: relationships to socio-demographic and clinical characteristics. *Psychiatry Res.* (2011) 190:193–9. 10.1016/j.psychres.2011.05.016 21621853

[67] Allen MH Debanné M Lazignac C Adam E Dickinson LM Damsa C. Effect of nicotine replacement therapy on agitation in smokers with schizophrenia: a double-blind, randomized, placebo-controlled study. *Am J Psychiatry.* (2011) 168:395–9. 10.1176/appi.ajp.2010.10040569 21245085

[68] Schechter MA. Nicotine replacement therapy in the psychiatric emergency department. *Am J Psychiatry.* (2011) 168:347–9. 10.1176/appi.ajp.2010.10111680 21474593

